# *catena*-Poly[[hexa­kis(μ-4-methyl­benzoato)-κ^2^ *O*,*O*′;κ^15^ *O*,*O*′:*O*-trieuropium(III)]-tris­(μ-4-methyl­benzoato)-κ^2^ *O*,*O*′;κ^6^ *O*,*O*′:*O*]

**DOI:** 10.1107/S1600536808022836

**Published:** 2008-07-23

**Authors:** Chao-Hua Zhang, Peng-Zhi Hong, Wen-Dong Song

**Affiliations:** a School of Food Science and Technology, Guang Dong Ocean University, Zhan Jiang 524088, People’s Republic of China; b College of Science, Guang Dong Ocean University, Zhan Jiang 524088, People’s Republic of China

## Abstract

The title europium(III) carboxyl­ate, [Eu_3_(C_8_H_7_O_2_)_9_]_*n*_, has three independent Eu atoms, two of which are eight-coordinate in a square-anti­prismatic coordination geometry, whereas the third is nine-coordinate in a tricapped trigonal-prismatic coordination geometry. The metal atoms are linked by two bidentate and seven tridentate methyl­benzoate groups into a linear chain running along the *b*-axis direction.

## Related literature

For the crystal structures of metal complexes of 4-toluic acid, see: Song, Gu, Hao & Yan (2008 ▶); Song, Wang & Ji (2008 ▶); Song, Wang & Miao (2008 ▶); Song, Yan & Hao (2008 ▶).
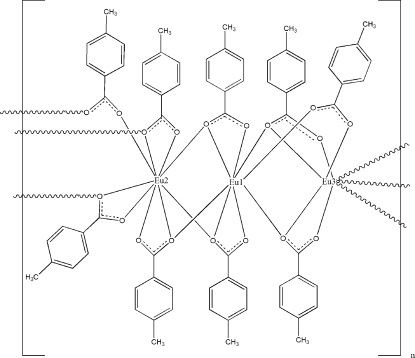


## Experimental

### 

#### Crystal data

[Eu_3_(C_8_H_7_O_2_)_9_]
*M* *_r_* = 1672.10
Monoclinic, 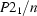

*a* = 13.8417 (4) Å
*b* = 22.4998 (7) Å
*c* = 21.8170 (7) Å
β = 96.490 (2)°
*V* = 6751.0 (4) Å^3^
*Z* = 4
Mo *K*α radiation
μ = 2.82 mm^−1^
*T* = 296 (2) K
0.25 × 0.21 × 0.20 mm

#### Data collection

Bruker APEXII area-detector diffractometer
Absorption correction: multi-scan (*SADABS*; Sheldrick, 1996 ▶) *T*_min_ = 0.539, *T*_max_ = 0.602 (expected range = 0.509–0.569)
67521 measured reflections
15508 independent reflections
11353 reflections with *I* > 2σ(*I*)
*R*_int_ = 0.073

#### Refinement

*R*[*F* ^2^ > 2σ(*F* ^2^)] = 0.042
*wR*(*F* ^2^) = 0.108
*S* = 1.06
15508 reflections
847 parameters
H-atom parameters constrained
Δρ_max_ = 0.79 e Å^−3^
Δρ_min_ = −1.64 e Å^−3^

### 

Data collection: *APEX2* (Bruker, 2004 ▶); cell refinement: *SAINT* (Bruker, 2004 ▶); data reduction: *SAINT*; program(s) used to solve structure: *SHELXS97* (Sheldrick, 2008 ▶); program(s) used to refine structure: *SHELXL97* (Sheldrick, 2008 ▶); molecular graphics: *SHELXTL* (Sheldrick, 2008 ▶); software used to prepare material for publication: *SHELXTL*.

## Supplementary Material

Crystal structure: contains datablocks I, global. DOI: 10.1107/S1600536808022836/ng2476sup1.cif

Structure factors: contains datablocks I. DOI: 10.1107/S1600536808022836/ng2476Isup2.hkl

Additional supplementary materials:  crystallographic information; 3D view; checkCIF report

## Figures and Tables

**Fig. 1.**
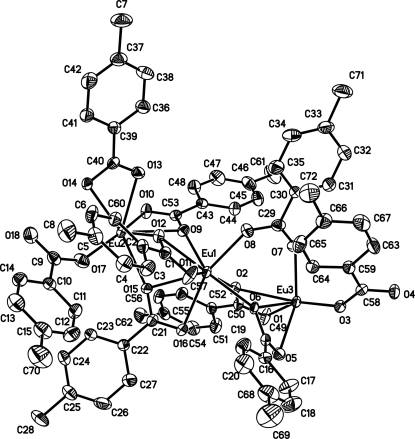
The molecular structure showing the atomic-numbering scheme and displacement ellipsoids drawn at the 30% probability level.

## supplementary crystallographic information

### Comment

As a building block, 4-methylbenzate ligand is an excellent candidate for the construction of supramolecular complexes (Song, Gu, Hao & Yan, 2008; Song, Wang & Ji, 2008; Song, Wang & Miao, 2008; Song, Yan & Hao, 2008).  Recently, we obtained the title coordination polymer by the hydrothermal reaction of Eu_2_O_3_, 4-methylbenzoic acid and HNO_3_ in water.

In the asymmetric unit of the title complex (I), there are three crystallographically independent Eu^III^ ions: one nine-coordinated Eu^III^ centre is coordinated by eight oxygen atoms from six 4-methylbenzoate ligands, and the other Eu^III^ centres are eight-coordinated by eight oxygen atoms from six 4-methylbenzoate ligands (Fig. 1). The 4-methylbenzoate ligands link the metal centres to form a polymeric chain (Fig. 1), in which the three unique Eu^III^ ions, separated by 3.738 (2), 3.906 (8) and 3.949 (7) Å, respectively, are in turn interconnected *via* the carboxylate groups of 4-methylbenzoate ligands.

### Experimental

A mixture of Eu_2_O_3_ (0.5 mmol), 4-methylbenzoic acid (0.5 mmol), HNO_3_ (0.15 ml) and H_2_O (10 ml) was placed in a 23 ml Teflon reactor, which was heated to 433 K for three days and then cooled to room temperature at a rate of 10 K h^-1^ to obtain block colorless crystals.

### Refinement

Carbon-bound H atoms were placed at calculated positions and were treated as riding on the parent C atoms with C—H = 0.93–0.96 Å, and with *U*_iso_(H) = 1.2 or 1.5 *U*_eq_(C).

### Crystal data

**Table:**

[Eu_3_(C_8_H_7_O_2_)_9_]	*F*_000_ = 3312
*M**_r_* = 1672.10	*D*_x_ = 1.645 Mg m^−^^3^
Monoclinic, *P*2_1_/*n*	Mo *K*α radiation λ = 0.71073 Å
Hall symbol: -P 2yn	Cell parameters from 9100 reflections
*a* = 13.8417 (4) Å	θ = 1.4–28.0º
*b* = 22.4998 (7) Å	µ = 2.82 mm^−^^1^
*c* = 21.8170 (7) Å	*T* = 296 (2) K
β = 96.490 (2)º	Block, colorless
*V* = 6751.0 (4) Å^3^	0.25 × 0.21 × 0.20 mm
*Z* = 4	

### Data collection

**Table:**

Bruker APEXII area-detector diffractometer	15508 independent reflections
Radiation source: fine-focus sealed tube	11353 reflections with *I* > 2σ(*I*)
Monochromator: graphite	*R*_int_ = 0.073
*T* = 296(2) K	θ_max_ = 27.5º
φ and ω scans	θ_min_ = 1.3º
Absorption correction: multi-scan(SADABS; Sheldrick, 1996)	*h* = −17→17
*T*_min_ = 0.539, *T*_max_ = 0.602	*k* = −29→29
67521 measured reflections	*l* = −28→26

### Refinement

**Table:**

Refinement on *F*^2^	Secondary atom site location: difference Fourier map
Least-squares matrix: full	Hydrogen site location: inferred from neighbouring sites
*R*[*F*^2^ > 2σ(*F*^2^)] = 0.042	H-atom parameters constrained
*wR*(*F*^2^) = 0.108	*w* = 1/[σ^2^(*F*_o_^2^) + (0.0443*P*)^2^ + 4.4935*P*] where *P* = (*F*_o_^2^ + 2*F*_c_^2^)/3
*S* = 1.06	(Δ/σ)_max_ = 0.002
15508 reflections	Δρ_max_ = 0.79 e Å^−^^3^
847 parameters	Δρ_min_ = −1.64 e Å^−^^3^
Primary atom site location: structure-invariant direct methods	Extinction correction: none

### Special details

**Table:**

Geometry. All e.s.d.'s (except the e.s.d. in the dihedral angle between two l.s. planes) are estimated using the full covariance matrix. The cell e.s.d.'s are taken into account individually in the estimation of e.s.d.'s in distances, angles and torsion angles; correlations between e.s.d.'s in cell parameters are only used when they are defined by crystal symmetry. An approximate (isotropic) treatment of cell e.s.d.'s is used for estimating e.s.d.'s involving l.s. planes.
Refinement. Refinement of *F*^2^ against ALL reflections. The weighted *R*-factor *wR* and goodness of fit *S* are based on *F*^2^, conventional *R*-factors *R* are based on *F*, with *F* set to zero for negative *F*^2^. The threshold expression of *F*^2^ > σ(*F*^2^) is used only for calculating *R*-factors(gt) *etc*. and is not relevant to the choice of reflections for refinement. *R*-factors based on *F*^2^ are statistically about twice as large as those based on *F*, and *R*- factors based on ALL data will be even larger.

### Fractional atomic coordinates and isotropic or equivalent isotropic displacement parameters (Å^2^)

**Table:**

	*x*	*y*	*z*	*U*_iso_*/*U*_eq_	
Eu1	0.204866 (18)	0.640991 (10)	0.239152 (11)	0.03031 (7)	
Eu2	0.247652 (17)	0.477164 (10)	0.251356 (11)	0.02904 (7)	
Eu3	0.282169 (18)	0.806685 (10)	0.279298 (11)	0.03000 (7)	
C1	0.0871 (4)	0.5670 (2)	0.1535 (2)	0.0349 (11)	
C2	0.0180 (4)	0.5315 (2)	0.1117 (2)	0.0373 (12)	
C3	−0.0730 (4)	0.5541 (3)	0.0921 (3)	0.0492 (14)	
H3	−0.0890	0.5924	0.1037	0.059*	
C4	−0.1403 (4)	0.5209 (3)	0.0556 (3)	0.0536 (15)	
H4	−0.2014	0.5367	0.0431	0.064*	
C5	−0.1178 (5)	0.4643 (3)	0.0374 (3)	0.0501 (15)	
C6	−0.0265 (5)	0.4420 (2)	0.0558 (3)	0.0514 (15)	
H6	−0.0105	0.4041	0.0433	0.062*	
C7	0.3894 (7)	0.3672 (4)	−0.1070 (3)	0.086 (2)	
H7A	0.4113	0.3268	−0.1086	0.129*	
H7B	0.4393	0.3935	−0.1181	0.129*	
H7C	0.3317	0.3724	−0.1353	0.129*	
C8	−0.1926 (5)	0.4269 (3)	−0.0015 (3)	0.074 (2)	
H8A	−0.2337	0.4074	0.0248	0.111*	
H8B	−0.1600	0.3977	−0.0238	0.111*	
H8C	−0.2313	0.4521	−0.0301	0.111*	
C9	0.0347 (4)	0.3962 (3)	0.2248 (3)	0.0440 (13)	
C10	−0.0702 (4)	0.4102 (2)	0.2115 (2)	0.0382 (12)	
C11	−0.1052 (5)	0.4660 (3)	0.2248 (3)	0.0521 (15)	
H11	−0.0646	0.4943	0.2452	0.063*	
C12	−0.2030 (5)	0.4784 (3)	0.2066 (3)	0.0656 (19)	
H12	−0.2273	0.5154	0.2159	0.079*	
C13	−0.2285 (4)	0.3826 (3)	0.1648 (3)	0.0613 (17)	
H13	−0.2699	0.3541	0.1455	0.074*	
C14	−0.1325 (4)	0.3685 (3)	0.1820 (3)	0.0477 (14)	
H14	−0.1095	0.3308	0.1739	0.057*	
C15	−0.2642 (4)	0.4382 (3)	0.1756 (3)	0.0598 (17)	
C16	−0.0177 (4)	0.7830 (2)	0.2281 (2)	0.0395 (12)	
C17	−0.0661 (5)	0.8349 (3)	0.2367 (3)	0.0678 (19)	
H17	−0.0365	0.8639	0.2627	0.081*	
C18	−0.1579 (6)	0.8445 (4)	0.2074 (4)	0.078 (2)	
H18	−0.1894	0.8801	0.2134	0.094*	
C19	−0.0620 (4)	0.7407 (3)	0.1889 (3)	0.0547 (16)	
H19	−0.0294	0.7058	0.1815	0.066*	
C20	−0.1542 (5)	0.7502 (4)	0.1608 (3)	0.072 (2)	
H20	−0.1842	0.7208	0.1354	0.087*	
C21	0.0982 (4)	0.5835 (2)	0.3264 (2)	0.0373 (11)	
C22	0.0341 (4)	0.5525 (2)	0.3654 (2)	0.0370 (11)	
C23	0.0410 (5)	0.4923 (3)	0.3761 (3)	0.0486 (14)	
H23	0.0889	0.4705	0.3595	0.058*	
C24	−0.0221 (5)	0.4639 (3)	0.4108 (3)	0.0588 (17)	
H24	−0.0169	0.4231	0.4172	0.071*	
C25	−0.0932 (5)	0.4955 (3)	0.4364 (3)	0.0547 (16)	
C26	−0.0985 (5)	0.5558 (3)	0.4267 (3)	0.0682 (19)	
H26	−0.1455	0.5777	0.4440	0.082*	
C27	−0.0362 (4)	0.5844 (3)	0.3921 (3)	0.0573 (16)	
H27	−0.0409	0.6253	0.3865	0.069*	
C28	−0.1626 (5)	0.4640 (4)	0.4741 (3)	0.079 (2)	
H28A	−0.1904	0.4922	0.5001	0.119*	
H28B	−0.1281	0.4341	0.4991	0.119*	
H28C	−0.2135	0.4456	0.4469	0.119*	
C29	0.3319 (4)	0.7273 (3)	0.1543 (2)	0.0426 (13)	
C30	0.3748 (4)	0.7327 (2)	0.0949 (2)	0.0359 (11)	
C31	0.4263 (5)	0.7827 (3)	0.0815 (3)	0.0541 (15)	
H31	0.4338	0.8140	0.1095	0.065*	
C32	0.4671 (5)	0.7866 (3)	0.0263 (3)	0.0694 (19)	
H32	0.5011	0.8207	0.0176	0.083*	
C33	0.4578 (5)	0.7410 (3)	−0.0155 (3)	0.0600 (17)	
C34	0.4053 (5)	0.6922 (3)	−0.0018 (3)	0.0654 (19)	
H34	0.3970	0.6612	−0.0302	0.079*	
C35	0.3646 (4)	0.6876 (3)	0.0525 (3)	0.0557 (16)	
H35	0.3298	0.6536	0.0605	0.067*	
C36	0.3956 (4)	0.4401 (3)	0.0509 (3)	0.0510 (15)	
H36	0.4295	0.4706	0.0726	0.061*	
C37	0.3671 (5)	0.3816 (3)	−0.0418 (3)	0.0576 (16)	
C38	0.4155 (5)	0.4259 (3)	−0.0085 (3)	0.0620 (17)	
H38	0.4630	0.4473	−0.0259	0.074*	
C39	0.3260 (4)	0.4090 (2)	0.0775 (2)	0.0389 (12)	
C40	0.3049 (4)	0.4258 (2)	0.1406 (2)	0.0359 (11)	
C41	0.2774 (5)	0.3635 (3)	0.0448 (2)	0.0492 (14)	
H41	0.2304	0.3417	0.0622	0.059*	
C42	0.2991 (6)	0.3506 (3)	−0.0142 (3)	0.0641 (19)	
H42	0.2662	0.3196	−0.0358	0.077*	
C43	0.5085 (4)	0.5867 (2)	0.2799 (2)	0.0352 (11)	
C44	0.5100 (4)	0.6460 (2)	0.2636 (3)	0.0455 (13)	
H44	0.4524	0.6643	0.2475	0.055*	
C45	0.5941 (4)	0.6785 (3)	0.2707 (3)	0.0545 (16)	
H45	0.5927	0.7183	0.2592	0.065*	
C46	0.6804 (5)	0.6534 (3)	0.2945 (3)	0.0577 (17)	
C47	0.6805 (4)	0.5936 (3)	0.3102 (3)	0.0645 (18)	
H47	0.7385	0.5757	0.3262	0.077*	
C48	0.5969 (4)	0.5606 (3)	0.3028 (3)	0.0507 (15)	
H48	0.5989	0.5205	0.3131	0.061*	
C49	0.0825 (4)	0.7758 (2)	0.2578 (2)	0.0352 (11)	
C50	0.3291 (4)	0.7149 (2)	0.3703 (2)	0.0322 (10)	
C51	0.3793 (5)	0.6917 (3)	0.4805 (3)	0.0536 (16)	
H51	0.3812	0.7323	0.4886	0.064*	
C52	0.3513 (4)	0.6720 (2)	0.4208 (2)	0.0358 (11)	
C53	0.4184 (4)	0.5513 (2)	0.2748 (2)	0.0347 (11)	
C54	0.4040 (5)	0.6525 (3)	0.5276 (3)	0.0556 (16)	
H54	0.4216	0.6670	0.5672	0.067*	
C55	0.4032 (4)	0.5920 (3)	0.5173 (3)	0.0455 (14)	
C56	0.3745 (4)	0.5723 (2)	0.4583 (3)	0.0500 (14)	
H56	0.3728	0.5317	0.4503	0.060*	
C57	0.3482 (4)	0.6113 (2)	0.4109 (3)	0.0460 (13)	
H57	0.3281	0.5967	0.3716	0.055*	
C58	0.2101 (3)	0.8950 (2)	0.1485 (2)	0.0334 (11)	
C59	0.1816 (4)	0.8574 (2)	0.0935 (2)	0.0362 (11)	
C60	0.0417 (4)	0.4747 (2)	0.0924 (2)	0.0406 (12)	
H60	0.1032	0.4590	0.1042	0.049*	
C61	0.7732 (5)	0.6895 (4)	0.3054 (4)	0.095 (3)	
H61A	0.8253	0.6682	0.2899	0.142*	
H61B	0.7888	0.6964	0.3488	0.142*	
H61C	0.7642	0.7269	0.2844	0.142*	
C62	0.4348 (5)	0.5500 (3)	0.5699 (3)	0.071 (2)	
H62A	0.5042	0.5517	0.5793	0.106*	
H62B	0.4157	0.5102	0.5582	0.106*	
H62C	0.4046	0.5614	0.6056	0.106*	
C63	0.1914 (5)	0.8789 (3)	0.0356 (3)	0.0554 (16)	
H63	0.2149	0.9172	0.0310	0.066*	
C64	0.1458 (4)	0.8007 (2)	0.0991 (2)	0.0381 (12)	
H64	0.1378	0.7857	0.1379	0.046*	
C65	0.1218 (4)	0.7659 (3)	0.0473 (3)	0.0513 (15)	
H65	0.0969	0.7279	0.0517	0.062*	
C66	0.1344 (5)	0.7869 (3)	−0.0104 (3)	0.0521 (15)	
C67	0.1667 (6)	0.8441 (3)	−0.0159 (3)	0.0665 (19)	
H67	0.1721	0.8597	−0.0549	0.080*	
C68	−0.2037 (5)	0.8020 (4)	0.1691 (4)	0.075 (2)	
C69	−0.3064 (6)	0.8120 (4)	0.1374 (5)	0.112 (3)	
H69A	−0.3066	0.8078	0.0936	0.168*	
H69B	−0.3277	0.8512	0.1467	0.168*	
H69C	−0.3496	0.7832	0.1521	0.168*	
C70	−0.3693 (5)	0.4546 (4)	0.1534 (4)	0.101 (3)	
H70A	−0.4004	0.4697	0.1873	0.152*	
H70B	−0.4034	0.4199	0.1368	0.152*	
H70C	−0.3704	0.4845	0.1219	0.152*	
C71	0.5037 (7)	0.7442 (4)	−0.0751 (3)	0.096 (3)	
H71A	0.5535	0.7741	−0.0716	0.144*	
H71B	0.4549	0.7541	−0.1084	0.144*	
H71C	0.5318	0.7063	−0.0831	0.144*	
C72	0.1107 (6)	0.7477 (3)	−0.0677 (3)	0.080 (2)	
H72A	0.1645	0.7218	−0.0721	0.120*	
H72B	0.0988	0.7724	−0.1036	0.120*	
H72C	0.0538	0.7244	−0.0632	0.120*	
O1	0.3381 (3)	0.76936 (15)	0.37999 (17)	0.0552 (11)	
O2	0.3002 (3)	0.69761 (14)	0.31548 (15)	0.0371 (8)	
O3	0.2092 (2)	0.87436 (14)	0.20319 (14)	0.0344 (8)	
O4	0.2347 (3)	0.94765 (15)	0.14131 (16)	0.0436 (9)	
O5	0.1154 (3)	0.80769 (16)	0.30228 (17)	0.0436 (9)	
O6	0.1398 (2)	0.73859 (13)	0.23484 (15)	0.0326 (7)	
O7	0.3440 (3)	0.76835 (19)	0.19304 (17)	0.0528 (10)	
O8	0.2850 (3)	0.68048 (18)	0.16297 (19)	0.0537 (10)	
O9	0.3385 (2)	0.57569 (15)	0.25196 (15)	0.0364 (8)	
O10	0.4184 (3)	0.49851 (16)	0.29149 (18)	0.0465 (9)	
O11	0.0666 (3)	0.61844 (17)	0.1673 (2)	0.0595 (12)	
O12	0.1683 (2)	0.54461 (15)	0.17555 (15)	0.0362 (8)	
O13	0.3485 (3)	0.46824 (17)	0.16781 (18)	0.0501 (10)	
O14	0.2391 (2)	0.39781 (14)	0.16678 (15)	0.0356 (8)	
O15	0.1642 (3)	0.55402 (15)	0.30270 (16)	0.0409 (8)	
O16	0.0899 (3)	0.63751 (16)	0.3154 (2)	0.0588 (11)	
O17	0.0931 (3)	0.43705 (19)	0.24689 (18)	0.0520 (10)	
O18	0.0624 (3)	0.34538 (19)	0.2124 (2)	0.0587 (11)	

### Atomic displacement parameters (Å^2^)

**Table:**

	*U*^11^	*U*^22^	*U*^33^	*U*^12^	*U*^13^	*U*^23^
Eu1	0.03769 (14)	0.01972 (12)	0.03321 (14)	−0.00114 (9)	0.00260 (10)	−0.00083 (9)
Eu2	0.03426 (14)	0.02073 (12)	0.03163 (13)	−0.00024 (9)	0.00152 (10)	−0.00068 (9)
Eu3	0.03913 (14)	0.02113 (12)	0.02940 (13)	−0.00140 (10)	0.00231 (10)	0.00088 (9)
C1	0.039 (3)	0.032 (3)	0.033 (3)	0.001 (2)	0.002 (2)	0.001 (2)
C2	0.040 (3)	0.034 (3)	0.037 (3)	−0.006 (2)	0.000 (2)	0.001 (2)
C3	0.047 (3)	0.045 (3)	0.053 (4)	0.003 (3)	−0.005 (3)	−0.006 (3)
C4	0.042 (3)	0.058 (4)	0.058 (4)	−0.002 (3)	−0.006 (3)	−0.003 (3)
C5	0.058 (4)	0.055 (4)	0.035 (3)	−0.014 (3)	−0.004 (3)	0.001 (3)
C6	0.071 (4)	0.033 (3)	0.048 (3)	−0.012 (3)	−0.003 (3)	−0.006 (2)
C7	0.114 (7)	0.102 (6)	0.046 (4)	0.009 (5)	0.028 (4)	−0.015 (4)
C8	0.072 (5)	0.083 (5)	0.062 (4)	−0.032 (4)	−0.014 (4)	−0.012 (4)
C9	0.038 (3)	0.049 (3)	0.045 (3)	0.002 (3)	0.007 (2)	0.014 (3)
C10	0.036 (3)	0.043 (3)	0.036 (3)	0.000 (2)	0.008 (2)	0.005 (2)
C11	0.062 (4)	0.037 (3)	0.058 (4)	0.001 (3)	0.009 (3)	−0.005 (3)
C12	0.065 (4)	0.050 (4)	0.085 (5)	0.021 (3)	0.019 (4)	0.005 (3)
C13	0.045 (3)	0.074 (5)	0.062 (4)	−0.008 (3)	−0.006 (3)	0.000 (4)
C14	0.035 (3)	0.048 (3)	0.059 (4)	0.002 (2)	0.003 (3)	−0.006 (3)
C15	0.044 (3)	0.078 (5)	0.058 (4)	0.006 (3)	0.004 (3)	0.017 (3)
C16	0.038 (3)	0.039 (3)	0.043 (3)	0.005 (2)	0.011 (2)	−0.001 (2)
C17	0.070 (4)	0.067 (5)	0.066 (4)	0.024 (4)	0.005 (4)	−0.022 (4)
C18	0.073 (5)	0.083 (5)	0.078 (5)	0.041 (4)	0.005 (4)	−0.001 (4)
C19	0.044 (3)	0.047 (3)	0.070 (4)	0.007 (3)	−0.004 (3)	−0.002 (3)
C20	0.054 (4)	0.079 (5)	0.078 (5)	0.000 (4)	−0.014 (4)	−0.003 (4)
C21	0.045 (3)	0.030 (3)	0.037 (3)	0.003 (2)	0.003 (2)	0.000 (2)
C22	0.042 (3)	0.035 (3)	0.035 (3)	−0.002 (2)	0.008 (2)	−0.003 (2)
C23	0.061 (4)	0.041 (3)	0.048 (3)	−0.002 (3)	0.025 (3)	0.000 (3)
C24	0.069 (4)	0.050 (4)	0.062 (4)	−0.013 (3)	0.025 (3)	0.007 (3)
C25	0.051 (4)	0.072 (4)	0.043 (3)	−0.015 (3)	0.011 (3)	0.004 (3)
C26	0.054 (4)	0.083 (5)	0.073 (5)	0.013 (4)	0.032 (3)	0.007 (4)
C27	0.056 (4)	0.053 (4)	0.066 (4)	0.010 (3)	0.021 (3)	0.004 (3)
C28	0.065 (5)	0.107 (6)	0.072 (5)	−0.025 (4)	0.033 (4)	0.002 (4)
C29	0.041 (3)	0.049 (3)	0.038 (3)	0.012 (3)	0.006 (2)	0.004 (3)
C30	0.040 (3)	0.033 (3)	0.036 (3)	0.000 (2)	0.008 (2)	0.000 (2)
C31	0.070 (4)	0.044 (3)	0.051 (4)	−0.005 (3)	0.016 (3)	−0.007 (3)
C32	0.083 (5)	0.056 (4)	0.076 (5)	0.003 (4)	0.036 (4)	0.015 (4)
C33	0.070 (4)	0.063 (4)	0.051 (4)	0.026 (4)	0.021 (3)	0.006 (3)
C34	0.077 (5)	0.079 (5)	0.042 (4)	0.004 (4)	0.015 (3)	−0.018 (3)
C35	0.058 (4)	0.058 (4)	0.052 (4)	−0.010 (3)	0.008 (3)	−0.016 (3)
C36	0.051 (3)	0.058 (4)	0.046 (3)	−0.008 (3)	0.010 (3)	−0.006 (3)
C37	0.071 (4)	0.064 (4)	0.039 (3)	0.007 (3)	0.011 (3)	−0.004 (3)
C38	0.055 (4)	0.085 (5)	0.049 (4)	−0.005 (4)	0.019 (3)	−0.003 (3)
C39	0.041 (3)	0.041 (3)	0.034 (3)	0.005 (2)	0.004 (2)	0.005 (2)
C40	0.038 (3)	0.032 (3)	0.037 (3)	0.008 (2)	−0.001 (2)	−0.001 (2)
C41	0.072 (4)	0.044 (3)	0.032 (3)	−0.003 (3)	0.008 (3)	−0.003 (2)
C42	0.097 (5)	0.056 (4)	0.037 (3)	−0.008 (4)	0.000 (3)	−0.011 (3)
C43	0.036 (3)	0.040 (3)	0.030 (3)	0.001 (2)	0.005 (2)	−0.001 (2)
C44	0.049 (3)	0.039 (3)	0.048 (3)	−0.001 (2)	0.003 (3)	0.004 (3)
C45	0.047 (3)	0.051 (4)	0.066 (4)	−0.017 (3)	0.006 (3)	0.001 (3)
C46	0.048 (4)	0.076 (5)	0.049 (4)	−0.019 (3)	0.006 (3)	0.001 (3)
C47	0.039 (3)	0.083 (5)	0.069 (4)	−0.006 (3)	−0.006 (3)	0.003 (4)
C48	0.042 (3)	0.050 (4)	0.058 (4)	0.004 (3)	−0.004 (3)	0.008 (3)
C49	0.043 (3)	0.025 (2)	0.039 (3)	−0.001 (2)	0.010 (2)	0.003 (2)
C50	0.037 (3)	0.027 (2)	0.032 (3)	0.000 (2)	0.001 (2)	0.002 (2)
C51	0.082 (4)	0.040 (3)	0.036 (3)	−0.002 (3)	−0.004 (3)	0.002 (3)
C52	0.042 (3)	0.032 (3)	0.032 (3)	−0.003 (2)	0.002 (2)	0.002 (2)
C53	0.034 (3)	0.037 (3)	0.034 (3)	0.002 (2)	0.003 (2)	−0.005 (2)
C54	0.066 (4)	0.070 (4)	0.029 (3)	−0.004 (3)	−0.004 (3)	0.004 (3)
C55	0.042 (3)	0.049 (3)	0.045 (3)	0.000 (3)	0.004 (2)	0.019 (3)
C56	0.059 (4)	0.031 (3)	0.059 (4)	0.008 (3)	0.002 (3)	0.010 (3)
C57	0.061 (4)	0.037 (3)	0.040 (3)	0.007 (3)	0.004 (3)	0.003 (2)
C58	0.034 (3)	0.032 (3)	0.034 (3)	0.004 (2)	0.002 (2)	−0.002 (2)
C59	0.045 (3)	0.037 (3)	0.026 (2)	−0.001 (2)	0.001 (2)	0.001 (2)
C60	0.048 (3)	0.035 (3)	0.038 (3)	0.000 (2)	0.001 (2)	0.000 (2)
C61	0.054 (4)	0.117 (7)	0.113 (7)	−0.037 (5)	0.004 (4)	0.012 (6)
C62	0.076 (5)	0.074 (5)	0.063 (4)	0.012 (4)	0.010 (4)	0.036 (4)
C63	0.080 (4)	0.048 (3)	0.040 (3)	−0.020 (3)	0.015 (3)	0.000 (3)
C64	0.048 (3)	0.040 (3)	0.025 (2)	0.000 (2)	0.000 (2)	−0.001 (2)
C65	0.066 (4)	0.038 (3)	0.047 (3)	−0.004 (3)	−0.011 (3)	−0.006 (3)
C66	0.064 (4)	0.053 (4)	0.038 (3)	0.000 (3)	0.001 (3)	−0.009 (3)
C67	0.102 (6)	0.072 (5)	0.026 (3)	−0.006 (4)	0.012 (3)	0.000 (3)
C68	0.049 (4)	0.106 (7)	0.069 (5)	0.021 (4)	0.002 (3)	0.024 (4)
C69	0.060 (5)	0.136 (9)	0.131 (8)	0.033 (5)	−0.026 (5)	0.010 (6)
C70	0.052 (4)	0.129 (8)	0.121 (7)	0.018 (5)	0.000 (5)	0.038 (6)
C71	0.128 (8)	0.106 (7)	0.064 (5)	0.043 (6)	0.052 (5)	0.020 (5)
C72	0.118 (7)	0.081 (5)	0.040 (4)	−0.014 (5)	0.003 (4)	−0.024 (4)
O1	0.101 (3)	0.0233 (18)	0.036 (2)	−0.0015 (19)	−0.011 (2)	0.0008 (15)
O2	0.051 (2)	0.0318 (18)	0.0272 (18)	0.0022 (16)	−0.0034 (15)	−0.0006 (14)
O3	0.050 (2)	0.0314 (18)	0.0213 (16)	0.0030 (15)	0.0010 (14)	0.0004 (14)
O4	0.068 (3)	0.0286 (19)	0.034 (2)	−0.0071 (17)	0.0054 (17)	0.0010 (15)
O5	0.049 (2)	0.043 (2)	0.041 (2)	−0.0083 (17)	0.0111 (17)	−0.0092 (17)
O6	0.0441 (19)	0.0182 (15)	0.0361 (18)	−0.0010 (14)	0.0065 (15)	0.0013 (13)
O7	0.053 (2)	0.065 (3)	0.041 (2)	0.003 (2)	0.0085 (18)	−0.012 (2)
O8	0.063 (3)	0.046 (2)	0.055 (3)	0.001 (2)	0.022 (2)	0.0132 (19)
O9	0.0337 (18)	0.0331 (18)	0.041 (2)	0.0005 (15)	−0.0013 (15)	−0.0053 (15)
O10	0.046 (2)	0.035 (2)	0.057 (2)	−0.0033 (17)	−0.0047 (18)	0.0074 (18)
O11	0.060 (3)	0.032 (2)	0.080 (3)	0.0083 (18)	−0.023 (2)	−0.020 (2)
O12	0.0405 (19)	0.0297 (17)	0.0374 (19)	−0.0014 (15)	−0.0004 (15)	0.0016 (15)
O13	0.057 (2)	0.051 (2)	0.046 (2)	−0.0154 (19)	0.0192 (19)	−0.0147 (19)
O14	0.045 (2)	0.0278 (18)	0.0341 (18)	0.0008 (15)	0.0069 (15)	0.0048 (14)
O15	0.054 (2)	0.0317 (19)	0.041 (2)	0.0070 (17)	0.0220 (17)	0.0046 (15)
O16	0.076 (3)	0.028 (2)	0.081 (3)	0.0099 (19)	0.043 (2)	0.0059 (19)
O17	0.038 (2)	0.071 (3)	0.047 (2)	−0.015 (2)	0.0040 (17)	−0.006 (2)
O18	0.040 (2)	0.049 (3)	0.086 (3)	0.0065 (19)	0.007 (2)	0.014 (2)

### Geometric parameters (Å, °)

**Table:**

Eu1—O8	2.280 (4)	C29—C30	1.490 (7)
Eu1—O9	2.354 (3)	C30—C35	1.370 (7)
Eu1—O6	2.371 (3)	C30—C31	1.382 (8)
Eu1—O2	2.375 (3)	C31—C32	1.388 (9)
Eu1—O11	2.387 (4)	C31—H31	0.9300
Eu1—O16	2.429 (4)	C32—C33	1.371 (9)
Eu1—O15	2.499 (3)	C32—H32	0.9300
Eu1—O12	2.593 (3)	C33—C34	1.368 (10)
Eu2—O17	2.314 (4)	C33—C71	1.511 (9)
Eu2—O12	2.415 (3)	C34—C35	1.373 (9)
Eu2—O4^i^	2.420 (3)	C34—H34	0.9300
Eu2—O15	2.424 (3)	C35—H35	0.9300
Eu2—O13	2.426 (4)	C36—C39	1.371 (8)
Eu2—O10	2.473 (4)	C36—C38	1.391 (8)
Eu2—O9	2.548 (3)	C36—H36	0.9300
Eu2—O3^i^	2.561 (3)	C37—C38	1.365 (9)
Eu2—O14	2.561 (3)	C37—C42	1.364 (9)
Eu2—C58^i^	2.871 (5)	C38—H38	0.9300
Eu2—Eu3^i^	3.9074 (3)	C39—C41	1.378 (7)
Eu3—O18^ii^	2.309 (4)	C39—C40	1.488 (7)
Eu3—O7	2.320 (4)	C40—O13	1.244 (6)
Eu3—O3	2.391 (3)	C40—O14	1.292 (6)
Eu3—O1	2.397 (3)	C41—C42	1.385 (8)
Eu3—O14^ii^	2.398 (3)	C41—H41	0.9300
Eu3—O5	2.417 (4)	C42—H42	0.9300
Eu3—O2	2.582 (3)	C43—C44	1.383 (7)
Eu3—O6	2.596 (3)	C43—C48	1.396 (7)
Eu3—C9^ii^	3.247 (6)	C43—C53	1.474 (7)
Eu3—Eu2^ii^	3.9074 (3)	C44—C45	1.368 (8)
C1—O11	1.237 (6)	C44—H44	0.9300
C1—O12	1.275 (6)	C45—C46	1.369 (9)
C1—C2	1.479 (7)	C45—H45	0.9300
C2—C3	1.380 (7)	C46—C47	1.387 (9)
C2—C60	1.397 (7)	C46—C61	1.517 (9)
C3—C4	1.376 (8)	C47—C48	1.369 (8)
C3—H3	0.9300	C47—H47	0.9300
C4—C5	1.380 (8)	C48—H48	0.9300
C4—H4	0.9300	C49—O5	1.251 (6)
C5—C6	1.376 (9)	C49—O6	1.293 (6)
C5—C8	1.516 (8)	C50—O1	1.247 (6)
C6—C60	1.378 (7)	C50—O2	1.278 (5)
C6—H6	0.9300	C50—C52	1.471 (7)
C7—C37	1.522 (9)	C51—C54	1.367 (8)
C7—H7A	0.9600	C51—C52	1.389 (7)
C7—H7B	0.9600	C51—H51	0.9300
C7—H7C	0.9600	C52—C57	1.381 (7)
C8—H8A	0.9600	C53—O10	1.241 (6)
C8—H8B	0.9600	C53—O9	1.285 (6)
C8—H8C	0.9600	C54—C55	1.380 (8)
C9—O18	1.245 (7)	C54—H54	0.9300
C9—O17	1.281 (7)	C55—C56	1.378 (8)
C9—C10	1.481 (7)	C55—C62	1.512 (7)
C9—Eu3^i^	3.247 (6)	C56—C57	1.373 (7)
C10—C14	1.383 (7)	C56—H56	0.9300
C10—C11	1.388 (7)	C57—H57	0.9300
C11—C12	1.395 (9)	C58—O4	1.247 (6)
C11—H11	0.9300	C58—O3	1.282 (5)
C12—C15	1.365 (10)	C58—C59	1.484 (6)
C12—H12	0.9300	C58—Eu2^ii^	2.871 (5)
C13—C15	1.375 (9)	C59—C63	1.375 (7)
C13—C14	1.376 (8)	C59—C64	1.379 (7)
C13—H13	0.9300	C60—H60	0.9300
C14—H14	0.9300	C61—H61A	0.9600
C15—C70	1.526 (9)	C61—H61B	0.9600
C16—C17	1.369 (8)	C61—H61C	0.9600
C16—C19	1.376 (8)	C62—H62A	0.9600
C16—C49	1.472 (7)	C62—H62B	0.9600
C17—C18	1.374 (10)	C62—H62C	0.9600
C17—H17	0.9300	C63—C67	1.380 (8)
C18—C68	1.376 (11)	C63—H63	0.9300
C18—H18	0.9300	C64—C65	1.383 (7)
C19—C20	1.368 (8)	C64—H64	0.9300
C19—H19	0.9300	C65—C66	1.375 (8)
C20—C68	1.373 (10)	C65—H65	0.9300
C20—H20	0.9300	C66—C67	1.374 (9)
C21—O16	1.241 (6)	C66—C72	1.533 (8)
C21—O15	1.284 (6)	C67—H67	0.9300
C21—C22	1.473 (7)	C68—C69	1.526 (9)
C22—C23	1.375 (7)	C69—H69A	0.9600
C22—C27	1.390 (8)	C69—H69B	0.9600
C23—C24	1.378 (8)	C69—H69C	0.9600
C23—H23	0.9300	C70—H70A	0.9600
C24—C25	1.381 (9)	C70—H70B	0.9600
C24—H24	0.9300	C70—H70C	0.9600
C25—C26	1.373 (10)	C71—H71A	0.9600
C25—C28	1.510 (8)	C71—H71B	0.9600
C26—C27	1.368 (9)	C71—H71C	0.9600
C26—H26	0.9300	C72—H72A	0.9600
C27—H27	0.9300	C72—H72B	0.9600
C28—H28A	0.9600	C72—H72C	0.9600
C28—H28B	0.9600	O3—Eu2^ii^	2.561 (3)
C28—H28C	0.9600	O4—Eu2^ii^	2.420 (3)
C29—O7	1.250 (7)	O14—Eu3^i^	2.398 (3)
C29—O8	1.263 (7)	O18—Eu3^i^	2.309 (4)
			
O8—Eu1—O9	83.64 (13)	C24—C23—H23	119.6
O8—Eu1—O6	79.86 (13)	C23—C24—C25	120.6 (6)
O9—Eu1—O6	150.74 (11)	C23—C24—H24	119.7
O8—Eu1—O2	91.40 (14)	C25—C24—H24	119.7
O9—Eu1—O2	83.37 (11)	C26—C25—C24	118.3 (6)
O6—Eu1—O2	73.11 (11)	C26—C25—C28	121.5 (7)
O8—Eu1—O11	91.38 (16)	C24—C25—C28	120.2 (7)
O9—Eu1—O11	120.41 (12)	C27—C26—C25	121.5 (6)
O6—Eu1—O11	84.16 (12)	C27—C26—H26	119.2
O2—Eu1—O11	156.22 (12)	C25—C26—H26	119.2
O8—Eu1—O16	157.19 (13)	C26—C27—C22	120.2 (6)
O9—Eu1—O16	117.73 (13)	C26—C27—H27	119.9
O6—Eu1—O16	77.40 (12)	C22—C27—H27	119.9
O2—Eu1—O16	83.82 (14)	C25—C28—H28A	109.5
O11—Eu1—O16	84.50 (16)	C25—C28—H28B	109.5
O8—Eu1—O15	150.26 (13)	H28A—C28—H28B	109.5
O9—Eu1—O15	70.67 (12)	C25—C28—H28C	109.5
O6—Eu1—O15	129.70 (11)	H28A—C28—H28C	109.5
O2—Eu1—O15	99.98 (12)	H28B—C28—H28C	109.5
O11—Eu1—O15	89.06 (14)	O7—C29—O8	123.1 (5)
O16—Eu1—O15	52.32 (12)	O7—C29—C30	119.5 (5)
O8—Eu1—O12	91.01 (13)	O8—C29—C30	117.4 (5)
O9—Eu1—O12	69.20 (11)	C35—C30—C31	118.5 (5)
O6—Eu1—O12	134.66 (11)	C35—C30—C29	120.4 (5)
O2—Eu1—O12	152.01 (11)	C31—C30—C29	121.1 (5)
O11—Eu1—O12	51.49 (11)	C30—C31—C32	120.4 (6)
O16—Eu1—O12	103.64 (12)	C30—C31—H31	119.8
O15—Eu1—O12	66.38 (11)	C32—C31—H31	119.8
O17—Eu2—O12	82.49 (13)	C33—C32—C31	120.9 (7)
O17—Eu2—O4^i^	85.72 (14)	C33—C32—H32	119.6
O12—Eu2—O4^i^	145.42 (12)	C31—C32—H32	119.6
O17—Eu2—O15	79.09 (14)	C34—C33—C32	117.9 (6)
O12—Eu2—O15	70.39 (12)	C34—C33—C71	120.8 (7)
O4^i^—Eu2—O15	75.50 (12)	C32—C33—C71	121.4 (7)
O17—Eu2—O13	122.94 (13)	C33—C34—C35	122.0 (6)
O12—Eu2—O13	78.08 (13)	C33—C34—H34	119.0
O4^i^—Eu2—O13	134.49 (14)	C35—C34—H34	119.0
O15—Eu2—O13	138.70 (12)	C30—C35—C34	120.4 (6)
O17—Eu2—O10	158.87 (13)	C30—C35—H35	119.8
O12—Eu2—O10	117.58 (12)	C34—C35—H35	119.8
O4^i^—Eu2—O10	73.91 (13)	C39—C36—C38	119.9 (6)
O15—Eu2—O10	100.63 (13)	C39—C36—H36	120.0
O13—Eu2—O10	70.98 (13)	C38—C36—H36	120.0
O17—Eu2—O9	142.48 (13)	C38—C37—C42	117.3 (6)
O12—Eu2—O9	69.09 (11)	C38—C37—C7	120.9 (7)
O4^i^—Eu2—O9	103.77 (11)	C42—C37—C7	121.8 (6)
O15—Eu2—O9	68.77 (11)	C37—C38—C36	121.8 (6)
O13—Eu2—O9	75.54 (12)	C37—C38—H38	119.1
O10—Eu2—O9	51.52 (11)	C36—C38—H38	119.1
O17—Eu2—O3^i^	80.68 (13)	C36—C39—C41	119.1 (5)
O12—Eu2—O3^i^	154.27 (10)	C36—C39—C40	118.8 (5)
O4^i^—Eu2—O3^i^	51.95 (11)	C41—C39—C40	122.1 (5)
O15—Eu2—O3^i^	124.62 (11)	O13—C40—O14	119.4 (5)
O13—Eu2—O3^i^	95.01 (12)	O13—C40—C39	120.1 (5)
O10—Eu2—O3^i^	82.33 (11)	O14—C40—C39	120.5 (4)
O9—Eu2—O3^i^	133.67 (10)	C39—C41—C42	119.5 (6)
O17—Eu2—O14	74.37 (13)	C39—C41—H41	120.3
O12—Eu2—O14	87.96 (10)	C42—C41—H41	120.3
O4^i^—Eu2—O14	119.83 (11)	C37—C42—C41	122.4 (6)
O15—Eu2—O14	147.68 (12)	C37—C42—H42	118.8
O13—Eu2—O14	52.01 (12)	C41—C42—H42	118.8
O10—Eu2—O14	110.79 (12)	C44—C43—C48	117.4 (5)
O9—Eu2—O14	126.36 (11)	C44—C43—C53	122.7 (5)
O3^i^—Eu2—O14	68.83 (10)	C48—C43—C53	119.9 (5)
O17—Eu2—C58^i^	83.60 (14)	C45—C44—C43	121.5 (5)
O12—Eu2—C58^i^	164.41 (13)	C45—C44—H44	119.2
O4^i^—Eu2—C58^i^	25.48 (12)	C43—C44—H44	119.2
O15—Eu2—C58^i^	100.04 (13)	C44—C45—C46	121.2 (6)
O13—Eu2—C58^i^	115.69 (14)	C44—C45—H45	119.4
O10—Eu2—C58^i^	75.61 (13)	C46—C45—H45	119.4
O9—Eu2—C58^i^	119.89 (12)	C45—C46—C47	118.1 (6)
O3^i^—Eu2—C58^i^	26.51 (12)	C45—C46—C61	121.7 (7)
O14—Eu2—C58^i^	95.06 (12)	C47—C46—C61	120.2 (6)
O17—Eu2—Eu3^i^	62.01 (11)	C48—C47—C46	121.2 (6)
O12—Eu2—Eu3^i^	117.93 (8)	C48—C47—H47	119.4
O4^i^—Eu2—Eu3^i^	83.88 (8)	C46—C47—H47	119.4
O15—Eu2—Eu3^i^	137.13 (8)	C47—C48—C43	120.6 (6)
O13—Eu2—Eu3^i^	81.30 (9)	C47—C48—H48	119.7
O10—Eu2—Eu3^i^	109.24 (9)	C43—C48—H48	119.7
O9—Eu2—Eu3^i^	153.82 (8)	O5—C49—O6	119.1 (5)
O3^i^—Eu2—Eu3^i^	36.39 (7)	O5—C49—C16	121.4 (5)
O14—Eu2—Eu3^i^	36.57 (7)	O6—C49—C16	119.3 (4)
C58^i^—Eu2—Eu3^i^	60.44 (10)	O1—C50—O2	118.2 (4)
O18^ii^—Eu3—O7	77.16 (15)	O1—C50—C52	120.6 (4)
O18^ii^—Eu3—O3	97.52 (14)	O2—C50—C52	121.2 (4)
O7—Eu3—O3	80.74 (13)	C54—C51—C52	121.2 (5)
O18^ii^—Eu3—O1	81.90 (16)	C54—C51—H51	119.4
O7—Eu3—O1	120.18 (14)	C52—C51—H51	119.4
O3—Eu3—O1	157.95 (12)	C57—C52—C51	117.5 (5)
O18^ii^—Eu3—O14^ii^	78.72 (13)	C57—C52—C50	122.1 (5)
O7—Eu3—O14^ii^	142.44 (14)	C51—C52—C50	120.3 (5)
O3—Eu3—O14^ii^	74.37 (11)	O10—C53—O9	119.6 (4)
O1—Eu3—O14^ii^	83.96 (11)	O10—C53—C43	121.6 (4)
O18^ii^—Eu3—O5	152.07 (13)	O9—C53—C43	118.8 (4)
O7—Eu3—O5	128.34 (13)	C51—C54—C55	121.2 (5)
O3—Eu3—O5	78.45 (12)	C51—C54—H54	119.4
O1—Eu3—O5	91.72 (15)	C55—C54—H54	119.4
O14^ii^—Eu3—O5	73.56 (12)	C56—C55—C54	117.6 (5)
O18^ii^—Eu3—O2	106.11 (13)	C56—C55—C62	122.4 (6)
O7—Eu3—O2	82.11 (13)	C54—C55—C62	119.9 (6)
O3—Eu3—O2	146.77 (11)	C57—C56—C55	121.5 (5)
O1—Eu3—O2	51.44 (11)	C57—C56—H56	119.2
O14^ii^—Eu3—O2	132.51 (11)	C55—C56—H56	119.2
O5—Eu3—O2	90.41 (12)	C56—C57—C52	120.8 (5)
O18^ii^—Eu3—O6	155.92 (13)	C56—C57—H57	119.6
O7—Eu3—O6	79.13 (13)	C52—C57—H57	119.6
O3—Eu3—O6	82.71 (10)	O4—C58—O3	119.5 (4)
O1—Eu3—O6	106.76 (12)	O4—C58—C59	119.4 (4)
O14^ii^—Eu3—O6	123.89 (11)	O3—C58—C59	121.0 (4)
O5—Eu3—O6	51.73 (11)	O4—C58—Eu2^ii^	56.6 (2)
O2—Eu3—O6	66.19 (10)	O3—C58—Eu2^ii^	63.1 (2)
O18^ii^—Eu3—C9^ii^	17.20 (15)	C59—C58—Eu2^ii^	174.0 (3)
O7—Eu3—C9^ii^	81.23 (14)	C63—C59—C64	118.7 (5)
O3—Eu3—C9^ii^	81.71 (13)	C63—C59—C58	119.8 (5)
O1—Eu3—C9^ii^	94.04 (14)	C64—C59—C58	121.5 (4)
O14^ii^—Eu3—C9^ii^	67.84 (13)	C6—C60—C2	119.6 (5)
O5—Eu3—C9^ii^	140.07 (13)	C6—C60—H60	120.2
O2—Eu3—C9^ii^	123.32 (13)	C2—C60—H60	120.2
O6—Eu3—C9^ii^	156.62 (12)	C46—C61—H61A	109.5
O18^ii^—Eu3—Eu2^ii^	73.90 (11)	C46—C61—H61B	109.5
O7—Eu3—Eu2^ii^	105.52 (11)	H61A—C61—H61B	109.5
O3—Eu3—Eu2^ii^	39.45 (8)	C46—C61—H61C	109.5
O1—Eu3—Eu2^ii^	121.18 (8)	H61A—C61—H61C	109.5
O14^ii^—Eu3—Eu2^ii^	39.50 (8)	H61B—C61—H61C	109.5
O5—Eu3—Eu2^ii^	86.64 (9)	C55—C62—H62A	109.5
O2—Eu3—Eu2^ii^	172.01 (7)	C55—C62—H62B	109.5
O6—Eu3—Eu2^ii^	117.03 (7)	H62A—C62—H62B	109.5
C9^ii^—Eu3—Eu2^ii^	56.91 (10)	C55—C62—H62C	109.5
O11—C1—O12	119.6 (5)	H62A—C62—H62C	109.5
O11—C1—C2	120.3 (5)	H62B—C62—H62C	109.5
O12—C1—C2	120.1 (4)	C59—C63—C67	120.5 (6)
C3—C2—C60	118.7 (5)	C59—C63—H63	119.7
C3—C2—C1	119.8 (5)	C67—C63—H63	119.7
C60—C2—C1	121.5 (5)	C59—C64—C65	120.5 (5)
C4—C3—C2	120.9 (5)	C59—C64—H64	119.7
C4—C3—H3	119.6	C65—C64—H64	119.7
C2—C3—H3	119.6	C66—C65—C64	120.7 (6)
C3—C4—C5	120.6 (6)	C66—C65—H65	119.7
C3—C4—H4	119.7	C64—C65—H65	119.7
C5—C4—H4	119.7	C67—C66—C65	118.6 (5)
C6—C5—C4	118.8 (5)	C67—C66—C72	120.6 (6)
C6—C5—C8	120.5 (6)	C65—C66—C72	120.8 (6)
C4—C5—C8	120.7 (6)	C66—C67—C63	120.9 (6)
C5—C6—C60	121.4 (5)	C66—C67—H67	119.6
C5—C6—H6	119.3	C63—C67—H67	119.6
C60—C6—H6	119.3	C20—C68—C18	117.8 (6)
C37—C7—H7A	109.5	C20—C68—C69	121.3 (8)
C37—C7—H7B	109.5	C18—C68—C69	120.9 (8)
H7A—C7—H7B	109.5	C68—C69—H69A	109.5
C37—C7—H7C	109.5	C68—C69—H69B	109.5
H7A—C7—H7C	109.5	H69A—C69—H69B	109.5
H7B—C7—H7C	109.5	C68—C69—H69C	109.5
C5—C8—H8A	109.5	H69A—C69—H69C	109.5
C5—C8—H8B	109.5	H69B—C69—H69C	109.5
H8A—C8—H8B	109.5	C15—C70—H70A	109.5
C5—C8—H8C	109.5	C15—C70—H70B	109.5
H8A—C8—H8C	109.5	H70A—C70—H70B	109.5
H8B—C8—H8C	109.5	C15—C70—H70C	109.5
O18—C9—O17	122.9 (5)	H70A—C70—H70C	109.5
O18—C9—C10	118.1 (5)	H70B—C70—H70C	109.5
O17—C9—C10	118.9 (5)	C33—C71—H71A	109.5
O18—C9—Eu3^i^	33.3 (3)	C33—C71—H71B	109.5
O17—C9—Eu3^i^	89.7 (3)	H71A—C71—H71B	109.5
C10—C9—Eu3^i^	151.3 (4)	C33—C71—H71C	109.5
C14—C10—C11	119.8 (5)	H71A—C71—H71C	109.5
C14—C10—C9	119.5 (5)	H71B—C71—H71C	109.5
C11—C10—C9	120.6 (5)	C66—C72—H72A	109.5
C10—C11—C12	118.2 (6)	C66—C72—H72B	109.5
C10—C11—H11	120.9	H72A—C72—H72B	109.5
C12—C11—H11	120.9	C66—C72—H72C	109.5
C15—C12—C11	122.5 (6)	H72A—C72—H72C	109.5
C15—C12—H12	118.8	H72B—C72—H72C	109.5
C11—C12—H12	118.8	C50—O1—Eu3	100.0 (3)
C15—C13—C14	121.3 (6)	C50—O2—Eu1	155.5 (3)
C15—C13—H13	119.4	C50—O2—Eu3	90.3 (3)
C14—C13—H13	119.4	Eu1—O2—Eu3	105.58 (11)
C13—C14—C10	120.1 (6)	C58—O3—Eu3	146.0 (3)
C13—C14—H14	119.9	C58—O3—Eu2^ii^	90.4 (3)
C10—C14—H14	119.9	Eu3—O3—Eu2^ii^	104.16 (11)
C12—C15—C13	118.1 (6)	C58—O4—Eu2^ii^	97.9 (3)
C12—C15—C70	121.0 (7)	C49—O5—Eu3	96.1 (3)
C13—C15—C70	120.9 (7)	C49—O6—Eu1	146.2 (3)
C17—C16—C19	119.0 (5)	C49—O6—Eu3	87.0 (3)
C17—C16—C49	119.0 (5)	Eu1—O6—Eu3	105.25 (12)
C19—C16—C49	121.9 (5)	C29—O7—Eu3	142.1 (4)
C16—C17—C18	120.7 (7)	C29—O8—Eu1	137.3 (4)
C16—C17—H17	119.6	C53—O9—Eu1	160.7 (3)
C18—C17—H17	119.6	C53—O9—Eu2	91.9 (3)
C17—C18—C68	120.8 (7)	Eu1—O9—Eu2	99.29 (12)
C17—C18—H18	119.6	C53—O10—Eu2	96.5 (3)
C68—C18—H18	119.6	C1—O11—Eu1	99.8 (3)
C20—C19—C16	119.8 (6)	C1—O12—Eu2	145.1 (3)
C20—C19—H19	120.1	C1—O12—Eu1	89.0 (3)
C16—C19—H19	120.1	Eu2—O12—Eu1	96.48 (11)
C19—C20—C68	121.9 (7)	C40—O13—Eu2	97.7 (3)
C19—C20—H20	119.1	C40—O14—Eu3^i^	140.0 (3)
C68—C20—H20	119.1	C40—O14—Eu2	90.1 (3)
O16—C21—O15	118.8 (5)	Eu3^i^—O14—Eu2	103.94 (12)
O16—C21—C22	121.9 (5)	C21—O15—Eu2	162.4 (3)
O15—C21—C22	119.3 (4)	C21—O15—Eu1	91.9 (3)
C23—C22—C27	118.5 (5)	Eu2—O15—Eu1	98.80 (12)
C23—C22—C21	122.0 (5)	C21—O16—Eu1	96.4 (3)
C27—C22—C21	119.6 (5)	C9—O17—Eu2	146.8 (4)
C22—C23—C24	120.9 (6)	C9—O18—Eu3^i^	129.5 (4)
C22—C23—H23	119.6		

Symmetry codes: (i) −*x*+1/2, *y*−1/2, −*z*+1/2; (ii) −*x*+1/2, *y*+1/2, −*z*+1/2.
